# Potential Health Risk Associated with Mycotoxins in Oat Grains Consumed in Spain

**DOI:** 10.3390/toxins13060421

**Published:** 2021-06-13

**Authors:** Andrea Tarazona, José Vicente Gómez, Fernando Mateo, Misericordia Jiménez, Eva María Mateo

**Affiliations:** 1 Department of Microbiology and Ecology, University of Valencia, 46100 Burjasot, Valencia, Spain; Andrea.tarazona@uv.es (A.T.); J.vicente.gomez@uv.es (J.V.G.); Misericordia.jimenez@uv.es (M.J.); 2 Department of Electronic Engineering, ETSE, University of Valencia, 46100 Burjasot, Valencia, Spain; Fernando.mateo@uv.es; 3 Department of Microbiology, School of Medicine, 46010 Burjasot, Valencia, Spain

**Keywords:** mycotoxins, oats, co-occurrence, food safety, deoxynivalenol, zearalenone, HT-2 and T-2 toxins, aflatoxins, UPLC-MS/MS

## Abstract

Spain is a relevant producer of oats (*Avena sativa*), but to date there has been no study on the occurrence/co-occurrence of mycotoxins in oats marketed in Spain. The present study is addressed to overcome this lack of knowledge. One hundred oat kernel samples were acquired across different Spanish geographic regions during the years 2015–2019 and analyzed for mycotoxin content using an ultra-high performance liquid chromatography electrospray ionization tandem mass spectrometry (UPLC–ESI–MS/MS) method and matrix-matched calibration. The focus was on the regulated mycotoxins although other relevant mycotoxins were considered. The percentage of incidence (levels ≥ limit of detection), mean and range (ng/g) of mycotoxins were as follows: zearalenone (66%, mean 39.1, range 28.1–153), HT-2 toxin (47%, mean 37.1, range 4.98–439), deoxynivalenol, (34%, mean 81.4, range 19.1–736), fumonisin B1 (29%, mean 157.5, range 63.2–217.4), and T-2 toxin, (24%, mean 49.9, range 12.3–321). Fumonisin B2, 3-acetyldeoxynivalenol, aflatoxins B1, B2, and G2, and ochratoxin A were also detected at low levels, but aflatoxin G1 was not. The maximum limits established by the European Commission for unprocessed oats were not exceeded, except for zearalenone (in one sample), and the sum of aflatoxins (in two samples). Mycotoxin co-occurrence at quantifiable levels in the same sample (two to five combinations) was found in 31% of samples. The most common mixtures were those of HT-2 + T-2 toxins alone or together with deoxynivalenol and/or zearalenone.

## 1. Introduction

Oats (*Avena sativa* L.) belong to the grass family (Poaceae) and are a cereal that contains fiber (about 10–12%). Beta-glucans are the main components of the soluble dietary fiber, and they are prebiotic promoting the activity of beneficial microorganisms residing in the colon. These compounds are potentially beneficial to alleviate diabetes and could reduce hyperglycemia, hyperlipidemia, and hypertension. They promote wound healing and alleviate ischemic heart disease [1,2]. This cereal contains phytochemicals with antioxidant activity, unsaturated fatty acids, vitamins C and E, is high in protein and does not contain gluten. Thus, oat-containing foods are accepted well by people with celiac disease or gluten sensitivity. As a consequence, there has been increased consumer appreciation of the health-promoting benefits of this cereal and its by-products usually consumed as breakfast cereals characterized by its good taste, dietetic properties, and high beta-glucan content [3]. Moreover, oats has been traditionally used as a common livestock feed. This cereal grows in cool-weather areas of Northern and Central Europe, Russia, Canada, USA, or Australia, among other countries. In 2018, Spain produced more than 1.48 million tons of oats (being the largest producer in the European Union (EU) and the third largest producer in the world, behind Russia and Canada) [4,5]. Spain is both exporter and importer of oats; in 2018, oat exports exceeded imports of this cereal but in 2015–2017 and 2019 imports predominated [6].

Some fungal species can grow and produce mycotoxins in oats and other cereals, which constitutes a risk for human and animal health apart from deteriorating the product and being a huge problem for the market of such commodities. The most important mycotoxin-producing fungi in cereals are species belonging to the genera *Aspergillus*, *Fusarium*, *Penicillium*, and *Alternaria*. The most relevant mycotoxins are aflatoxins (AF), mainly aflatoxin B1 (AFB1), aflatoxin B2 (AFB2), aflatoxin G1 (AFG1), and aflatoxin G2 (AFG2), type B trichothecenes, such as deoxynivalenol (DON), 15-acetyldeoxynivalenol (15-ADON) and 3-acetyl-deoxynivalenol (3-ADON), and type A trichothecenes, such as T-2 toxin (T2) and HT-2 toxin (HT2), zearalenone (ZEA), fumonisins (FUM), mainly fumonisin B1 (FB1) and fumonisin B2 (FB2), and ochratoxin A (OTA). Mycotoxins can occur in cereal crops during pre-harvest and post-harvest during storage as a consequence of the development of toxicogenic species in these matrices under appropriate environmental conditions. The fungal species associated with AF production are mainly *Aspergillus flavus* (AFB1 and AFB2) and *A. parasiticus* (AFB1, AFB2, AFG1, and AFG2). OTA can be produced in cereals by *A. ochraceus*, *A. steynii*, *A. westerdijkiae* or some *Aspergillus* in section *Nigri*, or *Penicillium verrucosum* and *P. nordicum.* Fumonisins are associated generally with *Fusarium verticillioides* and *F. proliferatum*. The main species of the genus *Fusarium* associated with DON, 3-ADON and ZEA production are *F. graminearum*, *F. culmorum*, *F. equiseti* and *F. crookwellense.* HT2 and T2 are produced mainly by *F. sporotrichioides*, *F. langsethiae*, and *F. armeniacum* [7,8].

The occurrence of mycotoxins is oats has been surveyed in northern and central European countries, such as Norway [9], Sweden [10], Finland [11], UK [12], Ireland [13], Germany [14], Switzerland [15] or Denmark [16]. The levels of T2 and HT2 were higher in oats than in other cereals, like barley or wheat in Norway [9,17] or the UK [18,19], and production of these toxins in these countries was attributed to the prevalence of *F. langsethiae*. The European Commission has set maximum limits for certain mycotoxins in oats and other cereals [20]. As T2 and HT2 usually co-occurred and have similar toxicity, the sum of both has indicative limits in the European Union regulation of 1000 and 200 ng/g for hulled and de-hulled oats, respectively [21]. In the United States, contamination with OTA of oat-based food, even food for infants is a concerning issue while the degree of contamination in Europa is lower [22]. A review on mycotoxin occurrence in agricultural products and foodstuffs in Europe can be found in Luo et al. [23].

These mycotoxins have different individual toxicities. According to Eskola et al. [24] AF are amongst the most concerning substances; they are genotoxic, mutagen and carcinogenic being AFB1 the most toxic. FUM, especially FB1, can produce liver and kidney injury and are related to leukoencephalomalacia in horses and porcine pulmonary edema. Exposure to DON produces acute gastrointestinal symptoms in humans; in animals, the most common chronic effects from chronic exposure are suppression of weight gain and anorexia. Chronic exposure to T2 and HT2 in animals induces general toxicity, hemato- and immunotoxicity and high exposure produces vomiting. Although ZEA exhibits low acute toxicity, long-term exposure to this toxin is risky due to its potent estrogenic activity, being female pigs the most affected. OTA is nephrotoxic and a potent renal carcinogen in rodents; it affects mainly pigs, poultry and dogs, and has been classified as possibly carcinogenic to humans [24].

Another issue to take into account is the co-occurrence of various mycotoxins in the same oat sample due to possible interactions among them. The toxicity of mycotoxin mixtures is difficult to be foreseen based on their individual toxicities and exposure to these combinations can lead to additive, synergistic or antagonistic toxic effects [25,26].

Today most analytical methods to perform multi-mycotoxin analyses in cereal grains or cereal-based food/feed use tandem mass spectrometers (MS/MS) coupled through an interphase, such as electrospray ionization (ESI) or atmospheric pressure chemical ionization (APCI) sources, to high-performance liquid chromatographs or ultra-high performance liquid chromatographs (LC-MS/MS, UPLC-MS/MS) although there are problems concerning matrix effects, isobaric interference or lack of confidence in the identification of some contaminants [27,28,29].

To date there has been a lack of knowledge regarding the presence and distribution of mycotoxins in oats in Spain. Thirty samples of oat bran were found to have low contamination levels of DON, OTA, and ZEA and the co-occurrence of DON with ZEA was observed [30]. So far there have been no data on mycotoxin content in unprocessed oats marketed in Spain.

Therefore, the aim of the present study was to investigate the occurrence and co-occurrence of mycotoxins in oat grain samples taken in different places across Spain during a five-year period (2015–2019) using UPLC–MS/MS as the analytical technique to explore the potential health risk associated to oat consumption.

## 2. Results

### 2.1. Method Validation

**Figure 1:**
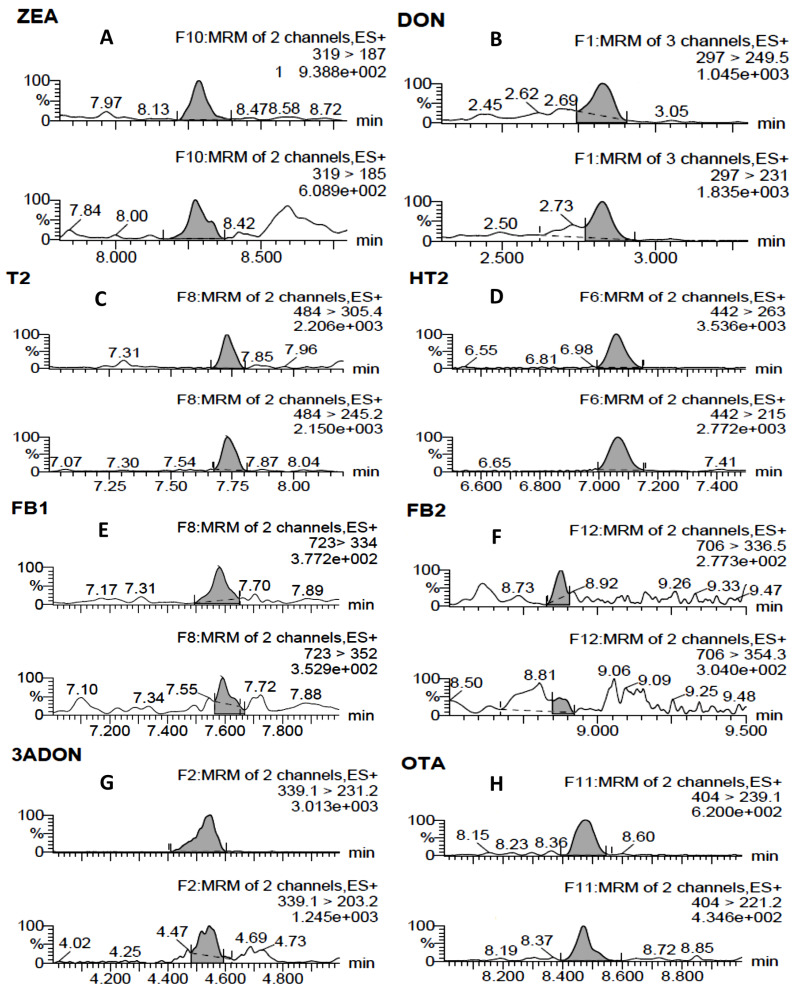
Ultra-high performance liquid chromatography electrospray ionization tandem mass spectrometry (UPLC–(ESI+)–MS/MS) multiple reaction monitoring (MRM) chromatograms of mycotoxins occurring in naturally contaminated oat grain samples. (**A**) ZEA (153 ng/g); (**B**) DON (307 ng/g); (**C**) T2 (12.3 ng/g); (**D**) HT2 (34.8 ng/g); (**E**) FB1 (200.7 ng/g); (**F**) FB2 (19 ng/g); (**G**) 3-ADON (37.9 ng/g); (**H**) OTA (2.24 ng/g). For each mycotoxin the upper chromatogram corresponds to the quantifier daughter ion (Q) and the lower one corresponds to the assistant qualifier daughter ion (q).

Table 1 lists, for the studied mycotoxins, the mean retention times, the limits of detection (LOD) and quantification (LOQ), the mean percentage of recoveries at four spiking levels of standards added to blank oat grain samples, and the mean relative standard deviation of recoveries under conditions of repeatability (RSD_r_). The area of the peak associated to the quantifier ion (Q) was used for quantification purposes while the presence of the peak associated to the assistant qualifier ion (q) was considered for confirmation. The retention time tolerance was 2.5%. The transitions used for the Q and q ions are those chosen in previous reports [28,31]. Some UPLC–(ESI+)–MS/MS multiple reaction monitoring (MRM) chromatograms of naturally contaminated oat grain samples are displayed in Figure 1.

### 2.2. Analysis of Oat Grain Samples

**Figure 2:**
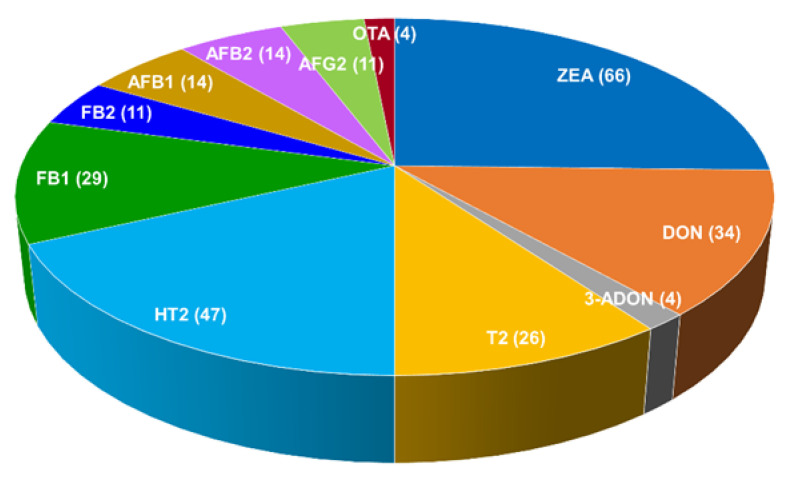
Number of oats grain samples having concentrations ≥LOD of the analyzed mycotoxins.

The summary of occurrence of the studied mycotoxins in 100 oat grain samples collected during the period 2015–2019 appears in Table 2. At least one mycotoxin was detected in 91 samples. The yearly distribution of the analyzed samples and the results related to the mycotoxins whose levels were ≥LOQ can be seen in Table 3. ZEA, HT2 and DON were the most abundant mycotoxins found. Most samples (64%) were analyzed in the years 2016–2017. The number of samples with mycotoxin content above the respective LOD is shown in Figure 2.

#### 2.2.1. Zearalenone

ZEA occurred at concentrations ≥LOQ in 37 samples and was detected in another 29 samples (Table 2). However, the range of quantifiable levels (28.1–153 ng/g) was not very wide. ZEA was found at levels >LOQ in the five years. The highest percentage occurred in 2016 (13 samples with levels ≥LOQ over 33 analyzed samples) (Table 3). A sample exceeded the EU ML set by the EC for this mycotoxin (100 ng/g) in year 2017.

#### 2.2.2. Deoxynivalenol (DON) and 3-Acetyl-Deoxynivalenol (3-ADON)

As can be seen in Table 2; Table 3, DON occurred in about one third of the analyzed samples. It was quantified in 22 samples and detected in other 12 samples (34% overall incidence). DON was found in all the five years of the study although the largest proportion of levels ≥LOQ (12/31) took place in 2017. The mean was 81.4 ng/g and the maximum level (736 ng/g) was below the EU ML (1750 ng/g). The mycotoxin 3-ADON was detected at levels ≥LOQ in only three samples in 2017 (Table 3) and was not separated from 15-ADON [28]. This mycotoxin was always found accompanied with DON.

#### 2.2.3. T-2 Toxin (T2) and HT-2 Toxin (HT2)

T2 and HT2 were detected in the oat samples analyzed. HT2 was detected in 47 samples and its levels were ≥LOQ in 36 samples. This toxin ranked second after ZEA in degree of incidence (Table 2). The sum of T2 and HT2 did not exceed the EU maximum recommended limit for unprocessed oats (1000 ng/g) [21]. Thirty-six samples were contaminated (>LOD) with T2 and its levels were usually lower than HT2 levels. These two type A trichothecenes were found at quantifiable levels in each of the five years under study (Table 3).

#### 2.2.4. Fumonisins

FB1 and FB2 were detected in 29% and 11% of the samples, respectively (Table 2). The yearly occurrence throughout 2015–2019 appears in Table 3. Six and three samples exhibited levels of FB1 and FB2 higher than their LOQ, respectively. The maximum levels of FB1 and FB2 were 217.4 and 64.0 ng/g, respectively. However, at present there is not a ML set for fumonisins in oats in the EU regulation.

#### 2.2.5. Aflatoxins

AFB1 and AFB2 were detected in 14 oat samples each, but AFB1 and AFB2 were quantified in five and one of them, respectively. The EU ML for AFB1 (2 ng/g) was not surpassed. Although AFG1 was not detected, AFG2 was detected in 11 samples and in two of them exceeded its LOQ in year 2017; moreover, these levels were so high that the sum of AF surpassed the EU ML of 4 ng/g. The mean and median for the sum of AF were 2.3 and 1.70 ng/g, respectively (Table 2). Then, 98% of oat samples were compliant with the EU regulation. The highest concentrations of AF were found in 2017. No AF was detected in the samples taken in 2015 and 2016 and only AFB1 was detected in 2018 and 2019 (Table 3).

#### 2.2.6. Ochratoxin A

OTA was detected in only 4% of samples and in only one sample the level (2.24 ng/g) was >LOQ in year 2017; thus, the EU ML of 5 ng/g was not surpassed (Table 2).

### 2.3. Co-Occurrence of Mycotoxins

**Figure 3:**
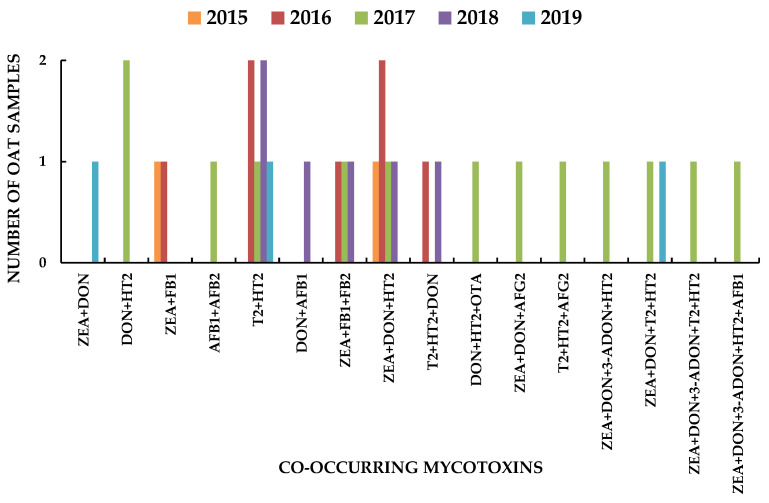
Co-occurrence of mycotoxins at levels ≥LOQ in oat kernel samples collected in Spain throughout the years 2015 to 2019.

Some mycotoxins co-occurred in the same sample. Figure 3 shows the number of oat samples where two or more (up to five) mycotoxins were present at levels ≥LOQ for every mycotoxin involved and the type of co-occurrence throughout the five years under study. Co-occurrence of the studied mycotoxins was observed in 31% of samples. The summarized data for the whole period are in Figure S1. T2 and HT2 co-occurred in 12 samples (the two alone in six samples and combined with other mycotoxins in another six). ZEA co-occurred with other mycotoxins in 17 samples. The combination of ZEA + DON was found in 12 samples, usually together other mycotoxins, even AF. The year when more samples showed co-occurring mycotoxins was 2017 (13/31 samples, 42%). This year two samples exceeded the EU ML for the sum of AF because of AFG2, and another sample exceeded the EU ML for ZEA (Table 2; Table 3). In 2018, 37.5% of samples showed co-occurring mycotoxins. There were six types of binary combination in 13 samples, six types of ternary combinations in 13 samples also, two types of quaternary combinations in three samples and two types of quinary combinations in two samples (Figure 3). The most common combinations were those of trichothecenes or trichothecenes with ZEA (24/31) although other mycotoxins also co-occurred in these mixtures. The proportion of samples where mycotoxins co-occurred at levels above their LOD was 67% and the number of combinations was higher.

## 3. Discussion

Concerning the analytical methodology used in the present study, the mean recovery rates were in the range of 85.3–100.4%, and the RSD_r_ of the recoveries ranged from 4.5% to 8.7%. They were considered acceptable and both fulfilled the performance criteria established in [32]. The LOQ values are below the EU regulatory or recommended limits in oats for all the analytes under study. No clean-up apart from physical centrifugation/filtration of the extracts was performed and the ESI was operated in positive mode.

The occurrence of mycotoxins in unprocessed oat grain marketed in Spain has not been previously reported, which hinders the comparison of the results obtained in this study with others in this country. From the results of the present study it may be deduced that, despite the high number of samples encompassing mycotoxin incidence, only one sample of oats contained a ZEA level surpassing the EU ML of 100 ng/g and two samples showed that their sum of AF exceeded the EU ML of 4 ng/g. Thus, only 3% of non-compliant samples with EU regulations [20] were found.

ZEA was detected in 66% of samples surveyed in our study, showing the highest mycotoxin incidence. Occurrence of ZEA, a mycotoxin produced by some *Fusarium* species, has been reported in some cereals and food in developed and developing countries [33,34,35,36,37,38,39]. ZEA levels detected in oats harvested in Ireland and UK were very low and the number of affected oats samples was scarce [13,38]. However, in Norway and Finland, ZEA has been found in oats reaching high maximum levels [9,23,35]. In Sweden, the incidence was variable from undetectable to high levels depending on the year and the region [10,23,35]. ZEA was not detected in oats surveyed in The Netherlands, but the number of samples in these countries was low [35]. In Swiss and Croatian oats, ZEA contamination was generally low [13,23]. In India, 32.5% of surveyed oats contained ZEA [34]. It seems that the incidence of ZEA is higher in oats marketed in Spain than in UK or Ireland although maximum levels in Scandinavian countries or Finland are reported to be much higher. ZEA exhibits low acute toxicity, but long-term exposure to this mycotoxin may pose a health risk. Estimates of dietary exposure suggest that current exposure levels to ZEA can be close to the tolerable daily intake for this toxin established in 0.25 μg/kg body weight [24,33,37].

The incidence of DON in oats marketed in Spain in the period 2015–2019 was 34%, which is a bit lower than the incidence found in maize in this country during the same period (40%) [31]. DON has been reported to occur in oats in many European countries like Ireland, Croatia, Hungary, Finland, Sweden, UK, Poland, Slovakia or Spain [13,23,33,40,41] but also in other countries around the world [39,41]. In the UK, DON was an occasional contaminant of oats and the maximum level was 1866 ng/g [38]. The incidence and maximum level recorded in Ireland was also low [13]. In contrast, high incidence rate and high levels of DON in oats were found in Norway and Sweden [41]; also very high levels of DON were found in Finland (mean 2690 ng/g; max. level 23,800 ng/g) with 32% samples exceeding the EU ML [11,23,41]. Thus, in Scandinavia and Finland, high DON concentrations in oats are a particular problem. Such a degree of contamination is much higher than that found in UK, Irish and Spanish oats (present study). In center Europe, the degree of DON incidence and its levels in oats changed with the localization and the year [39]. In Slovakia, DON occurrence in oats has been reported to be 30% [40]. The content of 3-ADON showed a strong correlation to DON concentrations [11]. In Canada, DON was detected most often in oats than in wheat or rye [42].

The predominance of HT2 over T2 in naturally contaminated oats and the levels of these trichothecenes observed in the present study (mean = 51.8 ng/g; max. concentration for T2 + HT2 = 760 ng/g) (Table 1) generally agree with previous reports [23,35,38,43] but there are large differences among countries. The problem of oat grain contamination with these trichothecenes is of concern in Europe. Thus, most samples surveyed in the UK showed HT2 and T2 levels >10 ng/g [38]. HT2 was the most frequently detected *Fusarium* mycotoxin in oats, and usually presented the highest concentration in some samples surpassing the maximum EU recommended limit. In Sweden, HT2 was found in 76–63% of the oat samples, while the respective incidence of T2 was 64–63%. The maximum concentrations were 571 ng/g (HT2) and 185 ng/g (T2) [10]. In Norway, HT2 and T2 occurred in 67% and 88%, respectively, of the oat samples surveyed and the highest levels were 2040 and 864 ng/g [9] and the recommended EU limit for the sum of the levels of HT2 and T2 was surpassed in 6% of the samples. Also in Finland these toxins reached high levels in oats [11,23]. Processing of oats drastically reduces mycotoxin contamination as most mycotoxins remain in the husk [44]. These two toxins were found in Polish oats [45], although their incidence was rather variable depending on the weather. T2 and HT2 were the major mycotoxins in oats collected in Switzerland, during 2013–2015 and the ML recommended by the EU for the sum of both toxins was exceeded in all the years [15]. In Croatia and Hungary, these mycotoxins were also found in oats at relatively low levels [23,46]. The problem of the high incidence and content of these trichothecenes in oats in Europe (mainly in Northern Europe) is not worrying in Canada, the largest exporter of oats in the world (incidence rate 7%) [42,47]. On the basis of the present study, the occurrence profile of T2/HT2 in unprocessed Spanish oats is different (lower concentrations) from the profile shown in UK, Ireland or other North European countries.

The relatively low occurrence and content of FB1 and FB2 in Spanish oats found in the present study contrasts with the high levels found in maize collected in Spain during the same period [31], but this fact is in agreement with most surveys as the occurrence of these mycotoxins in oats is rarely reported [23,48]. In fact, the EU has not set a ML for these mycotoxins in oats, in spite of their high toxicity. FB1 was found in a single hulless oat sample at the level of 19 ng/g in the Czech Republic [49]. Three oat samples from Malaysian markets were contaminated with FB1 and one sample contained FB2 [50], which agrees quite well with the results found in the present study. FB1 occurrence in Canadian oats was rare with one sample exhibiting a concentration of 79.2 ng/g [47]. These mycotoxins were not detected in oats grown in North European countries [8]. In view of the low levels found for these toxins in the analyzed oat samples collected in Spain and the worldwide status the associated health risk appears not to be worrying.

Considering the results of the present study, the health risk due to AF contamination of oat grain in Spain can be considered low (only 2% of samples exceeded the EU ML of 4 ng/g for the sum of AF), which is in agreement with other reports [30,47,51]. On the other hand, oat kernels from organic and conventional farming systems were analyzed in Poland and AF were detected in 50% and 26% of samples from organic and conventional farming, respectively, although levels were always <4 ng/g [45]. AF occurred in raw oat grain in 8 of 15 samples in a range of 0.12–1.94 ng/g [22]. Oats is not a cereal prone to contamination with AF [52].

Presence of OTA in oats in Spain seems not to be a concerning issue as deduced from the present report as it was detected in only 4% of samples, always below the EU ML. It was not detected in 98 maize samples collected in Spain during the same five-year period [31]. This mycotoxin has not been detected or its level was below the EU ML in most cereal samples analyzed in South Korea [29]. However, the health risk of contamination of oats or oat by-products with OTA is not negligible in some countries like Canada where almost 47% of samples contained OTA levels >0.5 ng/g [53] or the United States, the largest importing country, where OTA was detected in 70% oat-based breakfast cereal samples and even in 59% of oat-based infant cereals at worrying levels [22].

Some mycotoxins were found to co-occur in the same sample at levels ≥LOQ in 31% of samples. There were mixtures of 2 to 5 mycotoxins. The most common combination (alone or combined with other mycotoxins) was that of T2 and HT2 (12% of samples), which is logical due to their biochemical relationship. In Spain, mycotoxin co-occurrence has been reported in wheat and oat-based bran (18%), maize (33.5%), pig feed (69%), and barley (67%) [30,31,54,55]. In oats, toxin co-occurrence has also been observed in other countries [13,14,15]. This is a worldwide issue in cereals or feed and the number of co-occurring mycotoxins can be higher if more mycotoxins were monitored [23,25]. Mycotoxin interactions may change their individual toxicity in an unpredictable way [25]. Today there are no regulations considering the mixtures of mycotoxins in food or feed, except for total AF, sum of FUM, or T2 + HT2. Because of co-exposure to contaminated food, several mycotoxins can co-occur in urine samples [23,25]. The combinations AF + FUM, DON + ZEA, AF + OTA, and FUM + ZEA in foods and feed are the most observed in the literature [39,56], but the type and degree of relevance of the combinations largely depends on the world region examined; in Europe, the mixtures of trichothecenes or trichothecenes and ZEA are the most usual. The most common combination in oats is that of DON + HT2/T2 [48], which agrees with our results as 13/31 combinations found in the present study contained at least two of these mycotoxins; this proportion is lower than the percentage observed for such combination in maize in Spain during the same period [31]. Another relevant mixture is that of DON + ZEA, which was found in 12 samples usually associated with other mycotoxins. Mycotoxin co-occurrence can be explained because most fungi can produce various mycotoxins concurrently, food commodities can be contaminated by several fungi simultaneously or in quick succession, and diets are usually composed of multiple grain sources [25].

Data on combined toxic effects of mycotoxins are limited; therefore, the health risk from simultaneous exposure is not well known. Most studies on mutual interactions among mycotoxins have been conducted using animal/human cell lines and usually binary mixtures have been assayed [25]. Interactive cytotoxicity between AFB1 and ZEA and DON has been demonstrated at low and high doses in porcine kidney cells. AFB1 + DON, AFB1 + OTA, and OTA + DON presented dose-dependent effect in intestinal Caco-2 and hepatic HipG2 cell lines, decreasing cell viability at higher concentration ratios [57]. For the combination DON + T2 antagonism was observed, with acute exposure on Chinese hamster CHO-K1 and monkey Vero cells. Synergistic cytotoxicity resulted from the combination of DON + 3-DON in porcine IPEC-1 and human Caco-2 or human gastric epithelial cells, particularly at low inhibitory levels [58,59]. Combinations of DON with other type B trichothecenes produced synergistic effects [58]. DON and FUM have toxicities on the intestinal tract through different mechanisms: DON inhibits protein synthesis and FUM disrupt sphingolipid biosynthesis, and different even contradictory effects have been reported when administered to experimental animals, depending on the dose and target cells/systems. Interactions between AFB1 + FB1 range from lack of interaction to synergistic effects [22]. Taking into account the complexity of the possible interactions among the mycotoxins and the possible presence of other mycotoxins and masked mycotoxins not covered in this study, the phenomenon of co-occurring mycotoxins in oats and other cereals and their derived food products is a serious issue that deserves to be monitored and studied in depth in future research work.

## 4. Conclusions

This is the first report on the incidence of mycotoxins in unprocessed oats performed in several regions of Spain and spans the five-year period 2015–2019. One hundred oat samples were collected and mycotoxins were extracted using acetonitrile/water/formic acid (80:19:1, *v*/*v*/*v*) as the extracting solvent. After centrifugation and filtration of the extract and without any additional clean-up step they were determined by a validated UPLC–(ESI+)–MS/MS method using matrix-matched calibration. ZEA, followed by HT2, DON, FB1 and T2, in this order, were the most frequently detected mycotoxins. AFB1, AFB2, AFG2, FB2, 3-ADON and OTA were also detected while AFG1 was undetected. One sample showed a level of ZEA that exceeded the maximum limit set by the European Commission and two samples surpassed the limit for the sum of AF. Some mycotoxins (two to five) co-occurred at quantifiable levels in the same sample in 31% of the samples, which might increase the health risk of the individual mycotoxins involved although further studies on this topic are needed to correctly assess the toxicological impact of such combinations.

## 5. Materials and Methods

### 5.1. Samples

One hundred samples of oat kernels without visible signs of mold growth were collected in the years 2015–2019 in 30 grain stores or silos located across different regions of Spain (Castilla-La Mancha, Castilla-Leon, Aragón, Andalucía, Madrid, Navarra, Extremadura, and Valencia) according to the procedures given in Commission Regulation (EC) No 401/2006 [59]. Oat grain origin was usually not well-known. At least 1 kg of grain was taken. Aggregated samples (about 0.5 kg after reduction) were analyzed as soon as they reached the laboratory or were stored at −20 °C until analysis. The number of samples taken each year is shown in Table 3.

### 5.2. Standards and Reagents

Mycotoxin standards of AFB1, AFB2, AFG1, AFG2, DON, 3-ADON, OTA, ZEA, FB1, FB2, HT2 and T2 were purchased from Sigma (Sigma–Aldrich, Alcobendas, Spain). Formic acid (98% ACS) was purchased from Panreac (Castellar del Vallés, Spain); ammonium formate (>99% for LC-MS) was from VWR International Eurolab S.L. (Llinars del Vallés, Spain). Acetonitrile (MeCN) and methanol were supplied by J.T. Baker (Deventer, The Netherlands). All solvents were LC grade. Pure water was obtained from a Milli-Q apparatus (Millipore, Billerica, MA, USA) and was used when water was required.

### 5.3. Determination of Mycotoxins

The analytical method described in [31] was followed taking into consideration the different cereal type. Briefly, stock solutions of standards (1 mg/mL) were solved in MeCN and stored at −20 °C. Working standards were prepared by dilution of the stock solutions with MeCN-water 75:25 (*v*/*v*) and used for calibration. Standard diluted solutions were prepared at different concentrations. Oat grain samples were milled and homogenized; then, 2 g were extracted with MeCN-water-formic acid (80:19:1, *v*/*v*/*v*) by shaking on orbital shaker (1 h). The extract was centrifuged, 2 mL of the supernatant was filtered through 0.22 μm polytetrafluoroethylene (PTFE), diluted 1:2 with the same solvent and an aliquot of the clear extract was injected into the UPLC–(ESI+)-MS/MS system. The used instrument was an ACQUITY UPLC system (Waters, Manchester, UK), equipped with an electrospray ionization interface used in positive mode (ESI+) and an ACQUITY TQD tandem quadrupole mass spectrometer (Waters Co., Milford, MA, USA). Separation was carried out in a reversed-phase ACQUITY UPLC BEH C18 column (50 × 2.1 mm, 1.7 μm particle size) (Waters). The chromatographic conditions for separation and mass detection by multiple reaction monitoring (MRM), data acquisition and processing were as previously described [31]. For mycotoxin identification, mean retention times (2.5% tolerance) and the two most intense MRM transitions of the parent compound were monitored and selected. The most intense peak was used for quantification and the other assistant peak was considered for identification. The optimized MS/MS conditions, the quantitative ions (Q) and assistant qualifier ions (q) used for each mycotoxin are listed in Table S1. Signal ratios of these ions were used as an additional confirmation criterion [32,60]. Slight differences between the slopes of calibration curves using solvent for standards and the standards added to blank oat extracts were observed. Thus, matrix-matched calibration was performed using blank oat samples whose clear extracts were added with mycotoxin standards. Calibration curves were obtained by weighted linear regression of the areas of the peaks given by the quantifier ion versus mycotoxin concentration. Linearity was assumed when R^2^ > 0.99 and residuals were <20%. Validation of the method including estimation of LOD and LOQ of each mycotoxin was carried out; accuracy and precision under repeatability conditions was performed by spiking experiments at different levels on milled blank oats. The identification of mycotoxins in samples was based on their retention time and on the presence of the selected quantifier (Q) and qualifier (q) ions in the MRM chromatogram. Concentrations were calculated by interpolation of the peak area corresponding to the Q ion in the calibration lines. Further dilution of extracts was needed if the signal exceeded the linear calibration range.

## Figures and Tables

**Table 1:** Mean retention times (tr), limits of detection (LOD) and quantification (LOQ), mean recoveries and mean relative standard deviations of recoveries under conditions of repeatability (RSDr) for mycotoxins added to blank oat kernels at four levels.

Mycotoxin ^1^	Mean tr (min)	LOD (ng/g)	LOQ (ng/g)	Mean Recovery (%)	Mean RSD_r_ (%)
FB1	7.66	20	60	90.2	5.6
FB2	9.10	14	41	92.6	4.7
DON	2.82	6.0	18	85.3	8.1
3-ADON	4.53	3.0	9	86.9	5.7
ZEA	8.28	9.3	28	95.8	5.9
AFB1	5.93	0.3	0.9	98.2	6.6
AFB2	5.70	0.2	0.6	100.4	5.5
AFG1	5.35	0.7	2.1	94.6	7.3
AFG2	5.07	1.0	3.0	98.0	6.5
T2	7.72	4.0	12	95.8	8.5
HT2	7.04	1.5	4.6	97.4	4.7
OTA	8.46	0.6	1.8	99.5	4.8

^1^ FB1: fumonisin B1; FB2: fumonisin B2; DON: deoxynivalenol; 3-ADON: 3-acetyl-deoxynivalenol; ZEA: zearalenone; AFB1: aflatoxin B1; AFB2: aflatoxin B2; AFG1: aflatoxin G1; AFG2: aflatoxin G2; T2: T-2 toxin; HT2: HT-2 toxin; OTA: ochratoxin A.

**Table 2:** Occurrence of mycotoxins in oat kernel samples and their levels ^1^.

Mycotoxin	No of Samples with Levels <LOD	No of Samples with Levels between LOD and LOQ	No of Samples with Levels ≥LOQ	Range of Levels ≥LOQ (ng/g)	Mean/Median ^2^ (ng/g)	EU ML (No of Samples Exceeding the EU ML) ^3^
ZEA	34	29	37	28.1–153	39.1/32.6	100 (1)
DON	66	12	22	19.1–736	81.4/30.5	1750 (0)
3-ADON ^4^	96	1	3	9.2–42.6	29.9/37.8	–
T2	74	14	12	12.3–321	49.9/16.3	–
HT2	53	11	36	4.98–439	37.1/25.55	–
T2 + HT2	–	–	–	4.98–760	51.8/29.8	1000 (0)
FB_1_	71	23	6	63.2–217.4	157.5/194	–
FB_2_	89	8	3	42.5–64.0	50.6/45.2	–
FB_1_ + FB_2_	–	–	–	70.0–251.4	182.8/209.0	–
AFB_1_	86	9	5	1.06–1.70	1.34/1.28	2 (0)
AFB_2_	86	13	1	0.61	0.61/0.61	–
AFG_1_	100	–	–	–	–	–
AFG_2_	89	9	2	4.5–4.7	4.6/4.6	–
Sum of AF	–	–	–	0.61–4.7	2.3/1.70	4 (2)
OTA	96	3	1	2.24	2.24/2.24	5 (0)

^1^ The number of analyzed samples is N = 100. ^2^ Mean and median of concentrations ≥LOQ. ^3^ EU ML: European Union maximum limits (ng/g). For T2 + HT2 this is an indicative limit for oats (with husk) [20,21]. ^4^ Levels of 3-ADON include those of 15-ADON.

**Table 3:** Yearly variation from 2015 to 2019 of mycotoxin contamination at levels ≥LOQ in samples of oat kernels marketed in Spain. AFG1 was not detected.

	Year				
	2015 (N ^1^ = 10)	2016 (N = 33)	2017 (N = 31)	2018 (N = 16)	2019 (N = 10)
Mycotoxin	n ^2^/Mean/Range ^3^	n/Mean/Range	n/Mean/Range	n/Mean/Range	n/Mean/Range
ZEA	8/31.6/29.1–34.9	13/31.8/28.9–34.8	9/58.8/28.1–153	4/33.8/31.9–35.5	3/39.0/32.8–45.5
DON	1/48.9	4/32.2/24.9–48.9	12/123.8/19.1–736	3/26.6/24.8–29.5	2/23.5/21.2–25.8
3-ADON	–	–	3/29.9/9.24–42.65	–	–
T2	–	3/13.85/12.3–16.3	4/103/13.6–321	3/15.5/12.9–18.7	2/15.4/12.4–18.3
HT2	1/13.5	9/23.3/10.5–34.8	11/74.0/5.15–439	8/26.1/5.45–40.8	7/14.8/4.98–29.3
T2 + HT2	1/13.5	9/26.5/10.5–42.3	11/111/5.15–760	8/31.9/5.45–59.45	7/19.2/4.98–43.4
FB1	1/217.4	2/203.6/200.7–206.4	2/128.7/70–187.4	1/63.2	–
FB2	–	1/45.2	1/64.0	1/42.5	–
FB1 + FB2	1/217.4	2/226.2/200.7–251.6	2/160.7/70–251.4	1/105.7	–
AFB_1_	–	–	2/1.49/1.28–1.70	2/1.30/1.06–1.54	1/1.12
AFB2	–	–	1/0.61	–	–
AFG2	–	–	2/4.6/4.50–4.7	–	–
Sum of AF	–	–	5/2.64/0.61–4.7	2/1.30/1.06–1.54	1/1.12
OTA	–	–	1/2.24	–	–

^1^ N: Number of analyzed oat kernel samples; ^2^ n: Number of samples with mycotoxin levels ≥LOQ; For sums: number of samples with all the added concentrations ≥LOQ; ^3^ Mean/Range of mycotoxin concentrations ≥LOQ (ng/g).

## Data Availability

The data presented in this study are available within the article or supplementary material.

## Supplementary Materials

The following are available online at https://www.mdpi.com/article/10.3390/toxins13060421/s1, Figure S1: Number of analyzed oat samples with co-occurrence of mycotoxins (all ≥LOQ) in the period 2015–2019 (left axis) and % of samples over the 31 samples where co-occurrence was detected (right axis), Table S1: Optimized MS/MS parameters, quantitative daughter ion (Q) and assistant qualifier daughter ion (q) used.
