# Supplementary material for: Potential Health Risk Associated with Mycotoxins in Oat Grains Consumed in Spain
Source: Toxins (Basel). 2021 Jun 13;13(6):421. doi: 10.3390/toxins13060421 (PMC8232004; doi:10.3390/toxins13060421)
Supplement: Supplementary file 1 [file toxins-13-00421-s001.zip › toxins-1248299-supplementary.pdf]

Supplementary Materials: Potential Health Risk Associated with Mycotoxins in Oat Grains Consumed in Spain

Andrea Tarazona, José Vicente Gómez, Fernando Mateo, Misericordia Jiménez and Eva María Mateo

Table S1. Optimized MS/MS parameters, quantitative daughter ion (Q) and assistant qualifier daughter ion (q) used.

Mycotoxin	Elemental formula	Base mass (Dalton)	Precursor ion (m/z) (Dalton)		Product ions (m/z) (Dalton)	Cone voltage (V)	Collision energy (eV)
ZEA	$C_{18}H_{22}O_5$	318.147	319.0	$[M + H]^+$	187.0 [1]	20	20
					185.0 [2]	20	25
DON	$C_{15}H_{20}O_6$	296.126	297.0	$[M + H]^+$	231.0 [1]	20	15
					249.5 [2]	15	10
3-ADON/ 15-ADON	$C_{17}H_{22}O_7$	338.137	339.1	$[M + H]^+$	231.0 [1]	35	15
					203.2 [2]	35	12
HT-2 toxin	$C_{22}H_{32}O_8$	424.210	442.0	$[M + NH_4]^+$	263.0 [1]	20	15
					215.0 [2]	20	15
T-2 toxin	$C_{24}H_{34}O_9$	466.5	484.0	$[M + NH_4]^+$	305.4 [1]	20	15
					245.2 [2]	20	15
FB_1	$C_{34}H_{59}NO_{15}$	721.388	723.0	$[M + H]^+$	334.0 [1]	50	40
					352.0 [2]	50	40
FB_2	$C_{34}H_{59}NO_{14}$	705.394	706.0	$[M + H]^+$	336.0 [1]	50	40
					354.3 [2]	50	40
AFB1	$C_{17}H_{12}O_6$	312.063	313.0	$[M + H]^+$	285.0 [1]	70	25
					241.0 [2]	70	35
AFB2	$C_{17}H_{14}O_6$	314.079	315.0	$[M + H]^+$	287.0 [1]	70	30
					259.0 [2]	70	25
AFG1	$C_{17}H_{12}O_7$	328.058	329.0	$[M + H]^+$	243.0 [1]	70	25
					283.0 [2]	70	25
AFG2	$C_{17}H_{14}O_7$	330.074	331.0	$[M + H]^+$	257.0 [1]	70	30
					285.2 [2]	70	30
OTA	$C_{20}H_{18}ClNO_6$	403.082	404.0	$[M + H]^+$	239.1 [1]	25	20
					221.2 [2]	40	30

[1]: Quantifier ion (Q); [2]: Assistant qualifier ion (q).

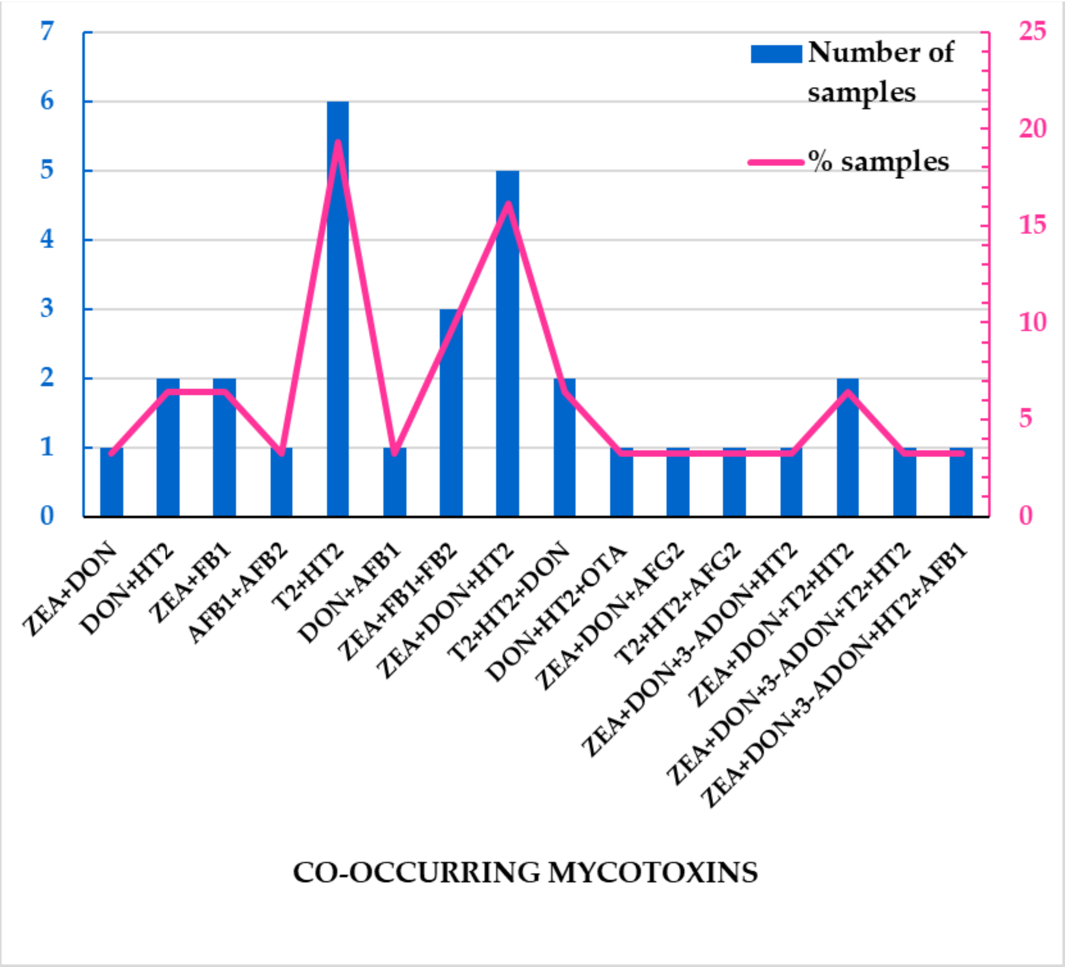

Figure S1. Number of analyzed oat samples with co-occurrence of mycotoxins (all ≥ LOQ) in the period 2015–2019 (left axis) and percentage of samples over the 31 samples where co-occurrence was detected (right axis).
